# A Novel Prognostic Index Model for Adult Hemophagocytic Lymphohistiocytosis: A Multicenter Retrospective Analysis in China

**DOI:** 10.3389/fimmu.2022.829878

**Published:** 2022-02-18

**Authors:** Ziyuan Shen, Yingliang Jin, Qian Sun, Shuo Zhang, Xi Chen, Lingling Hu, Chenlu He, Ying Wang, Qinhua Liu, Hao Zhang, Xin Liu, Ling Wang, Jun Jiao, Yuqing Miao, Weiying Gu, Fei Wang, Chunling Wang, Yuye Shi, Jingjing Ye, Taigang Zhu, Cai Sun, Xuguang Song, Linyan Xu, Dongmei Yan, Haiying Sun, Jiang Cao, Depeng Li, Zhenyu Li, Zhao Wang, Shuiping Huang, Kailin Xu, Wei Sang

**Affiliations:** ^1^ Department of Epidemiology and Biostatistics, School of Public Health, Xuzhou Medical University, Xuzhou, China; ^2^ Center for Medical Statistics and Data Analysis, School of Public Health, Xuzhou Medical University, Xuzhou, China; ^3^ Department of Hematology, Affiliated Hospital of Xuzhou Medical University, Xuzhou, China; ^4^ Department of Personnel, Suqian First Hospital, Suqian, China; ^5^ Department of Hematology, The First Affiliated Hospital of Anhui Medical University, Hefei, China; ^6^ Department of Hematology, The Affiliated Hospital of Jining Medical University, Jining, China; ^7^ Department of Hematology, Taian Central Hospital, Taian, China; ^8^ Department of Hematology, Yancheng First People’s Hospital, Yancheng, China; ^9^ Department of Hematology, The First People’s Hospital of Changzhou, Changzhou, China; ^10^ Department of Hematology, Huai’an First People’s Hospital, Huai’an, China; ^11^ Department of Hematology, Qilu Hospital of Shandong University, Jinan, China; ^12^ Department of Hematology, The General Hospital of Wanbei Coal-Electric Group, Suzhou, China; ^13^ Department of Hematology, Beijing Friendship Hospital, Capital Medical University, Beijing, China; ^14^ Huaihai Lymphoma Working Group: The Huaihai Lymphoma Working Group (HHLWG) was a non-governmental group established in November 2017 and included 18 medical centers in Huaihai Economic Zone of China, Xuzhou, China

**Keywords:** hemophagocytic lymphohistiocytosis, adult, multicenter, prognostic model, stratification

## Abstract

Hemophagocytic lymphohistiocytosis (HLH) is an immune disorder with rapid progression and poor survival. Individual treatment strategy is restricted, due to the absence of precise stratification criteria. In this multicenter retrospective study, we aimed to develop a feasible prognostic model for adult HLH in China. A total of 270 newly diagnosed patients of adult HLH were retrieved from the Huaihai Lymphoma Working Group (HHLWG), of whom 184 from 5 medical centers served as derivation cohort, and 86 cases from 3 other centers served as validation cohort. X-Tile program and Maxstat analysis were used to identify optimal cutoff points of continuous variables; univariate and multivariate Cox analyses were used for variable selection, and the Kaplan–Meier curve was used to analyze the value of variables on prognosis. The C-index, Brier Score, and calibration curve were used for model validation. Multivariate analysis showed that age, creatinine, albumin, platelet, lymphocyte ratio, and alanine aminotransferase were independent prognostic factors. By rounding up the hazard ratios from 6 significant variables, a maximum of 9 points was assigned. The final scoring model of HHLWG-HPI was identified with four risk groups: low risk (≤3 pts), low-intermediate risk (4 pts), high-intermediate risk (5-6 pts), and high risk (≥7 pts), with 5-year overall survival rates of 68.5%, 35.2%, 21.3%, and 10.8%, respectively. The C-indexes were 0.796 and 0.758 in the derivation and validation cohorts by using a bootstrap resampling program. In conclusion, the HHLWG-HPI model provides a feasible and accurate stratification system for individualized treatment strategy in adult HLH.

## Introduction

Hemophagocytic lymphohistiocytosis (HLH) is a severe hyperinflammatory syndrome characterized by excessive activation of T cells and macrophages and is classified into primary/hereditary (pHLH) and secondary/acquired (sHLH). Primary HLH is a fatal disease and usually develops in infancy or early childhood with a median survival of 2 months if without hematopoietic stem cell transplantation (1). Secondary HLH can be initiated by a large variety of inducements that activate the immune system, such as infections, autoimmune diseases, and tumors (2). Epstein–Barr virus is a common pathogenic factor of HLH, accounting for about 70% of infection-associated HLH (3, 4), and the most common cause of tumor-associated HLH is non-Hodgkin’s lymphoma (5, 6).

The widely used diagnostic criteria for HLH are HLH-2004 and HScore (7, 8). Currently, the recommended initial therapeutic regimens are HLH-94 and HLH-2004 (8, 9). Wang et al. proposed the DEP regimen as a salvage therapy, which showed an encouraging overall response rate (76.2%) in adult refractory and relapsed HLH (10). Due to the complexity of etiology and the heterogeneity of clinical manifestations, there is a lack of precise prognostic stratification and unified individualized treatment criteria in adult HLH.

In recent years, numerous studies have explored the prognostic factors of HLH in pediatric patients. Q. et al. proposed that the lymphocyte subset was essential for prognosis (11), and Pan et al. revealed that a higher disseminated intravascular coagulation (DIC) score and lower albumin, hemoglobin, and platelet levels were negative prognostic factors in malignancy-associated HLH (12). Schram et al. confirmed that platelets and alanine aminotransferase were independent factors for overall survival (OS) in adult HLH (13). Zhou et al. confirmed that high ferritin levels (>1,050 μg/l) were associated with poor survival (14). However, other studies revealed that ferritin was not an independent prognostic indicator for adult HLH. So, the value of ferritin on the prognosis of HLH was still controversial (15, 16). Furthermore, the heterogeneity and genetic abnormalities of HLH increase the difficulty of individualized treatment for adult HLH (5, 17, 18). Therefore, there is an urgent need to establish a prognostic stratification system for adult HLH.

Based on multicenter data from the Huaihai Lymphoma Working Group (HHLWG) in China, we carried out this retrospective study to explore the prognosis of adult HLH and attempted to establish a novel prognostic model to guide precise stratification for individualized treatment.

## Materials and Methods

### Patient Cohort

#### Data From Five Medical Centers of HHLWG for Prognostic Index Development

Data from five centers of HHLWG in this study served as the derivation cohort. The five centers are (1) Affiliated Hospital of Xuzhou Medical University (n = 75) (2), the Affiliated Hospital of Jining Medical University (n = 38) (3), Yancheng First People’s Hospital (n = 37) (4), Huai’an First People’s Hospital (n = 22), and (5) Taian Central Hospital (n = 12).

#### Data From Three Medical Centers of HHLWG for External Validation

**Figure 1:**
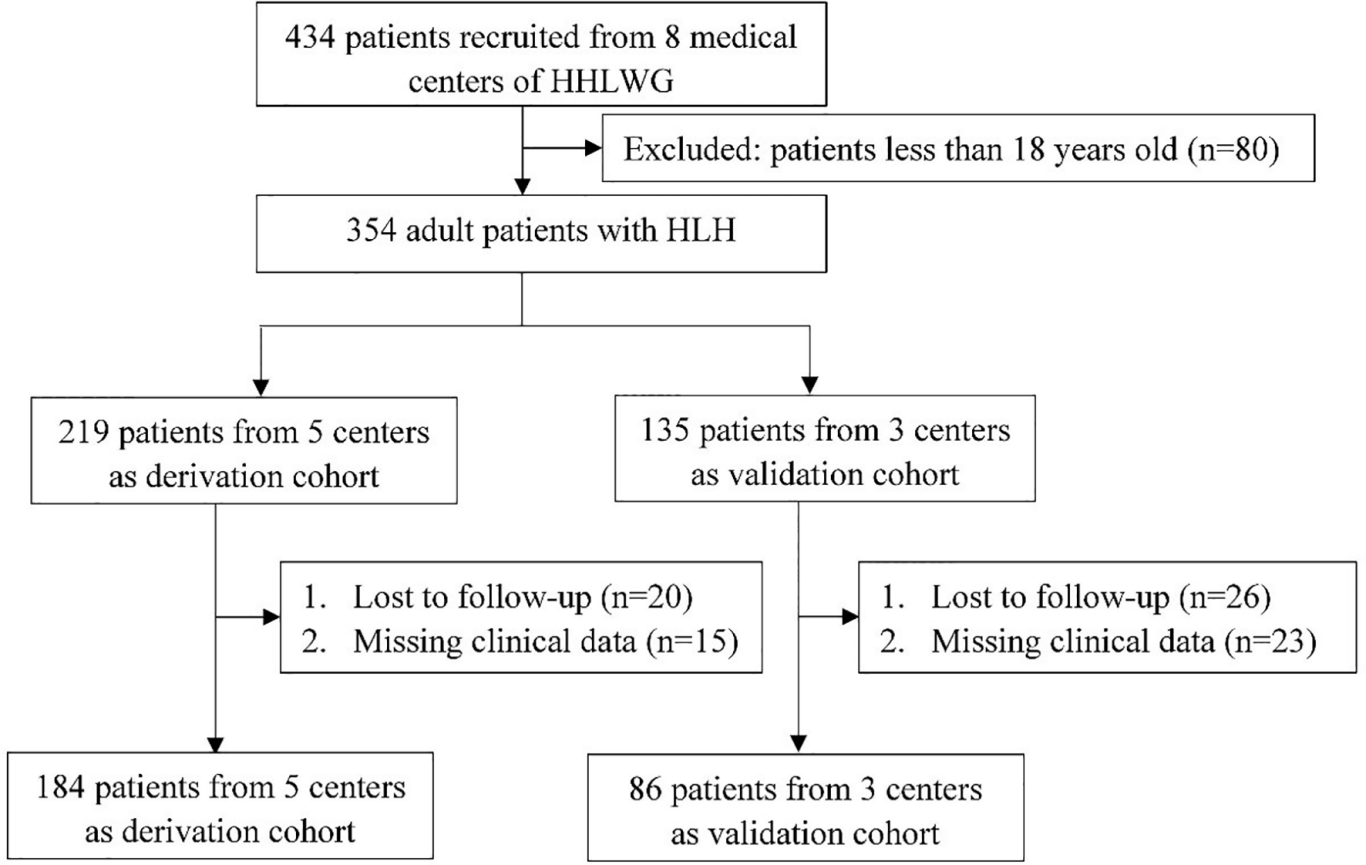
Flow chart of screening patients with HLH.

Data from three centers of HHLWG in this study served as the external validation cohort. The three centers are (1) The First Affiliated Hospital of Anhui Medical University (n = 40) (2), The First People’s Hospital of Changzhou (n = 30), and (3) Qilu Hospital of Shandong University (n = 16). Study approval was obtained from the independent Ethics Committees of each participating center in HHLWG and met the Helsinki Declaration. Patients over 18 years old with newly diagnosed HLH retrieved from the above centers between January 1, 2013, and August 19, 2020, were included. Median follow-up was 30.6 months [95% *CI* (22.2–38.9)] in the derivation cohort and 54.8 months [95% *CI* (25.3–84.3)] in the validation cohort. **Figure 1** shows the flowchart of the inclusion and exclusion processes in this study.

#### Baseline Characteristics of Patients

At admission, the following variables were collected: age, gender, etiologies, ferritin, triglycerides (TG), fibrinogen (FIB), lactate dehydrogenase (LDH), creatinine (Cr), alanine aminotransferase (ALT), hemoglobin (Hb), platelet (PLT), lymphocyte ratio (LYR), albumin (ALB), fever, EBV infection, presence or absence of splenomegaly, and therapeutic regimens.

#### Diagnosis of HLH

The diagnosis of HLH was established according to HLH-2004 diagnostic guidelines (8). Five of eight criteria are required to make a diagnosis of HLH (1): fever (2); splenomegaly (3); cytopenias (affecting ≥2 of 3 lineages in the peripheral blood: hemoglobin <90 g/l (in infants: hemoglobin < 100 g/l), platelets < 100 × 10^9^/l, neutrophils < 1.0 × 10^9^/ (4); hypofibrinogenemia and/or hypertriglyceridemia: fasting triglycerides ≥  3.0 mmol/l; fibrinogen ≤1.5 g/l) (5); hemophagocytosis in bone marrow, spleen, or lymph nodes (6); low or absent NK-cell activity (according to local laboratory reference) (7); ferritin ≥ 500 μg/l; and (8) soluble CD25 (soluble IL-2 receptor) ≥ 2,400 U/ml. All pathological biopsies were double blinded reviewed by at least two pathologists.

#### Follow-Up and Endpoints

Follow-up was conducted by consulting inpatient medical records and making phone calls. We followed up all the patients until February 19, 2021, or the death of patients. Overall survival (OS) was calculated as the interval between the time of diagnosis and death from any cause or the last follow-up. The survival status of all patients was confirmed with death records or a telephone call to next of kin (if patient died during the follow-up) or to the patients themselves.

### Statistical Analysis

#### Development of HHLWG-HPI

Based on the data from HHLWG, we attempted to develop a prognostic index model for adult HLH, the HHLWG-HPI. Data were presented as numbers (percentages) for categorical variables and median (interquartile range, IQR) for all continuous variables. Outliers were verified by the hospital medical record system. All cases were required to have complete clinical information in order to avoid unnecessary bias. The Shapiro–Wilk test was used to test the normality of numerical variables. Differences in clinical factors between etiology groups were analyzed by using the Kruskal–Wallis test and analysis of variance (ANOVA) test. Continuous variables were transformed into categorical variables by X-Tile program (Yale University, New Haven, CT, USA) (19) and Maxstat analysis (titled as Maximally Selected Rank Statistics). The X-Tile program can help divide patients into subgroups by determining the optimal cutoff points of a continuous or ordinal categorical variable based on the maximum *χ^2^* statistic value on the log-rank test (20). The Cox proportional hazard model was used to analyze the univariate association between prognostic factors and OS. All variables with *p*<0.1 in univariate analysis were kept in the multivariate analysis by using backward selection for the best predictor set, and Akaike information criteria (AIC) was used to evaluate the model. All statistical tests were two-sided, and the statistical significance was set at *p* < 0.05.

The prognostic index (HHLWG-HPI) was derived from the prognostic model that was identified using the Cox proportional hazards model. Index scores were assigned proportionally to the estimates of the relative contribution of the independent factors in the HHLWG-HPI model. The proximity of Kaplan–Meier was used to stratify patients into risk strata based on each score value.

#### Validation of HHLWG-HPI

The model was internally validated using a bootstrap resampling procedure (1,000 iterations) with a relatively corrected Harrell’s C-statistics (C-index). A C-index score around 0.70 indicates a good model (21). Brier Score is another score function that measures the accuracy of probabilistic prediction. In survival analysis, the Brier Score measures the mean of the difference between the observed and the estimated survival beyond a certain time. The score value ranges from 0 to 1, and a higher score indicates higher inaccuracy (22, 23). The calibration curve for probability of survival was used to show optimal agreement between prediction and actual observation. External validation of the HHLWG-HPI was performed using data from 3 medical centers of HHLWG. The C-index and calibration curve were based on the basis of the regression analysis. The statistical analysis was performed by SPSS statistics for Windows, Version 19.0 (Armonk, NY: IBM Corp.), and R software (version 4.0.3; http://www.Rproject.org).

## Results

### Cohort Characteristics

There were 434 HLH patients retrieved from HHLWG, of whom 354 were older than 18 years. Eighty-four patients were ineligible for inclusion, so 270 patients were eventually included in the whole cohort. The derivation cohort consisted of 184 cases, and the external validation cohort consisted of 86 patients. The characteristics of patients in both cohorts were compared, as shown in **Table 1**. The median age was 56 (IQR, 46–66) years, and 53.6% patients were male in the derivation cohort. Noticeably, patients in the derivation cohort were younger compared with the validation cohort (median age 56 vs. 61 years). Of the 184 patients, infections (n = 88) and tumor (n = 64) were the most frequent underlying etiologies, accounting for 83%. The results of the Kruskal–Wallis test showed that the lymphocyte ratio was significantly different among etiology groups, with the highest value in the infection-associated group.

**Table 1:** Basic clinical information of adult HLH.

Characteristics	Derivation cohort	Validation cohort	*p*
	n (%)	n (%)	
Age *^a^*	56 (46,66)	61 (47.75,70.00)	0.148
ALT *^a^*	72.5 (35.475,146.00)	77.5 (38.25,168.25)	0.736
Cr *^a^*	58.8 (45.15,77.75)	63.5 (50.00,80.00)	0.135
TG *^a^*	1.835 (1.418,2.455)	2.235 (1.795,3.050)	<0.050
Gender			0.088
Male	106 (57.60)	40 (46.51)	
Female	78 (42.40)	46 (53.49)	
Hb (g/L)			0.429
<120	156 (84.78)	76 (88.37)	
≥120	28 (15.22)	10 (11.63)	
PLT (×10^9^/L)			0.166
<100	138 (75.00)	71 (82.56)	
≥100	46 (25.00)	15 (17.44) 0)	
FIB (g/L)			0.115
<1.5	51 (27.72)	32 (37.21)	
≥1.5	133 (72.28)	54 (62.79)	
Splenomegaly			0.537
absence	67 (36.50)	28 (32.56)	
presence	117 (63.50)	58 (67.44)	

a Variables were presented using median and interquartile range.

ALT, alanine aminotransferase; Cr, creatinine; TG, triglycerides; Hb, hemoglobin; PLT, platelet; FIB, fibrinogen.

### Clinical Survival Analysis

**Figure 2:**
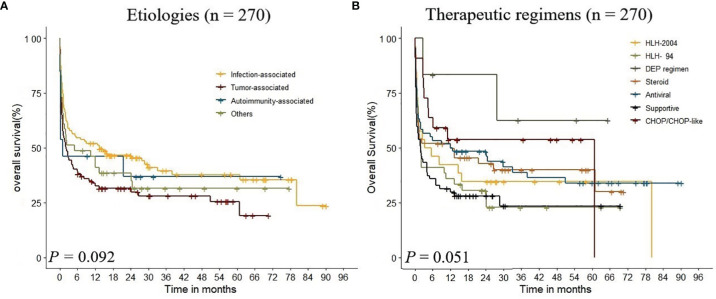
Kaplan–Meier analysis estimate of survival rate in adult HLH according to the underlying etiologies and therapeutic regimens.

In the whole cohort (n = 270), infection (n = 119) and tumor (n = 97) were the most frequent underlying etiologies, accounting for 80%. There was no significant difference among different etiology groups (*p* = 0.092, **Figure 2A**). The survival curves of different etiologies groups were examined by KM analysis, and the results showed that the OS of patients in the infection-associated group was significantly higher than that in the tumor-associated group (*χ^2^* = 6.400, *p* = 0.011), and there was no significant difference between the OS of patients in the infection-associated group and that in the autoimmunity-associated group (*χ^2^* = 0.895, *p* = 0.344). The results showed that the 5-year survival rates were significantly different in infection-associated, autoimmunity-associated, other etiologies, and tumor-associated groups (*p* < 0.01).

EBV-DNA data were available in 163 evaluable patients, of whom 82 exceeded 1,000 copies/ml. KM analysis showed that high EBV-DNA level was associated with poor outcome, with only 39% of 5-year OS. Maxstat analysis was performed in 105 cases with explicit value, and the optimal cutoff value of EBV-DNA was 1,520 copies/ml. The influence of different etiologies on the prognosis of adult HLH was further explored. According to KM analysis results, we found that there were no significant differences between EBV infection and other groups in prognosis (*p* > 0.05). In addition, we explored the prognostic value of ferritin in adult HLH. Of the 270 patients, the ferritin levels of 230 were above 500 ng/ml, and there was no survival difference between them and those with ferritin levels below 500 (*p* = 0.99). Of the 173 patients with explicit values that could be evaluated, an optimal cutoff value could not be obtained.

In this study, patients received regimens of HLH-94 (n = 39), HLH-2004 (n = 26), DEP regimen (n = 6), steroid (n = 50), antiviral (n = 60), CHOP/CHOP-like (n = 22), and supportive treatment (n = 67). The 1-year OS of each treatment group was 42.3%, 35.9%, 83.3%, 52.0%, 51.7%, 31.3%, and 53.7%, respectively. KM analysis indicated that there was no significant difference in therapeutic regimens on the prognosis of adult HLH (*p* = 0.051, **Figure 2B**). The results showed that the 5-year survival rates were significantly different among DEP regimen, HLH-94 regimen, HLH-2004 regimen, and steroid regimen groups (*p* < 0.01).

### Correlation Analysis of Immune Status and Survival

In patients with evaluable immune globulin (IgA, IgG, and IgM) and lymphocyte subset data, the correlations between immune status and survival were analyzed. The linear association of all immune factors was measured by Pearson’s correlation. The results suggested that CD8 had a significant positive linear association with IgG (*r* = 0.334, *p* = 0.015) and CD3 was negatively correlated with IgM (*r* = -0.383, *p* = 0.004).

**Figure 3:**
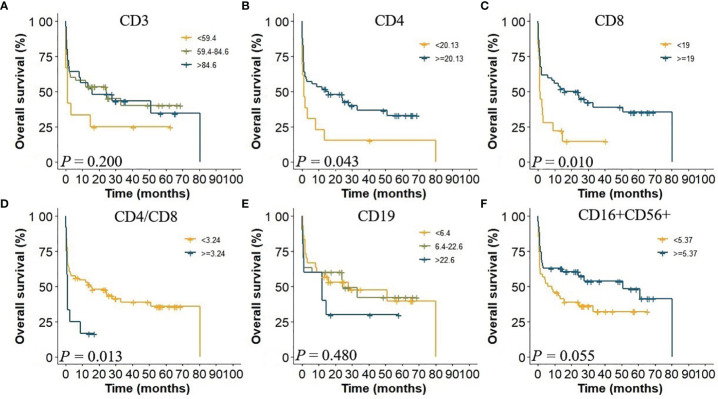
Kaplan–Meier analysis of lymphocyte subsets.

KM analysis indicated that there was no significant difference in immune globulin and lymphocyte subsets according to reference ranges (**Figure 3**). By Maxstat analysis, the optimal cutoff points for CD4+, CD8+ proportion, and CD4+/CD8+ ratio were 20.13, 19, and 3.24, respectively. Based on those cutoff points above, the survival of patients could be stratified.

### Cutoff Point Identification of Variables in the Derivation Cohort

**Figure 4:**
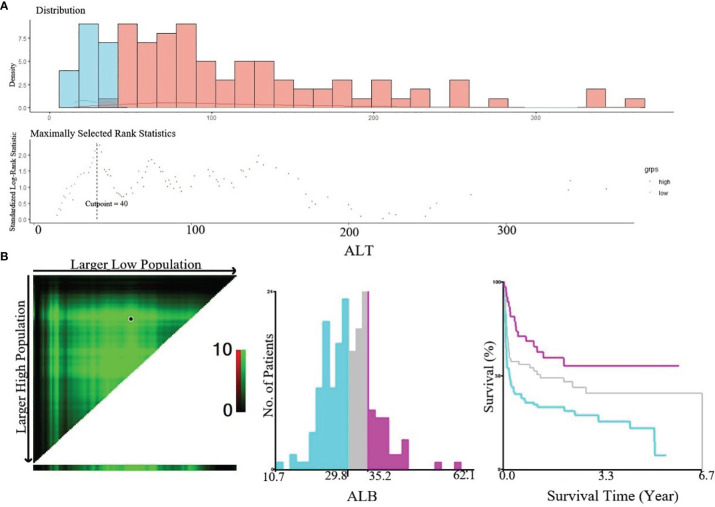
**(A)** Cut-off point of ALT defined by using maximally selected log-rank statistics. The estimated optimal cut-off point of ALT was 40 U/L; **(B)** X-Tile analysis of OS according to Alb. The black circles highlighted the optimal cut-off values which were presented in histograms.

In this study, continuous variables included in this study were age, lactate dehydrogenase (LDH), hemoglobin (Hb), platelet (PLT), fibrinogen (FIB), lymphocyte ratio (LYR), albumin (ALB), triglycerides (TG), creatinine (Cr), and alanine aminotransferase (ALT). The optimal cutoff values of TG, FIB, and ALT obtained by maximally selected rank statistics (**Figure 4A**) were 1.41, 1.2, and 40, respectively. Based on these cutoff values, we divided patients into higher group and lower group. X-Tile software was used to determine the optimal cutoff points for age, ALB, Hb, PLT, Cr, and LYR (**Figure 4B**). The best cutoff values for age were 50 and 65, for ALB 29.8 and 35.2, for Hb 68 and 104, for PLT 25 and 72, for Cr 53 and 66, and for LYR 21.1 and 45.8.

### Univariate and Multivariate Analyses of HLH Patients

The univariate analysis showed that age, Cr, ALB, PLT, LYR, Hb, FIB, ALT, etiologies, and gender significantly affected survival in the derivation cohort, whereas hepatosplenomegaly did not. Age, Cr, and ALB appeared to be stronger predictors (*p* < 0.001). The correlations between the clinical characteristics at diagnosis and OS are shown in **Table 2**.

**Table 2:** Univariate and multivariate analyses in the derivation cohort.

Univariate analysis			Multivariate analysis		
Variables	*p*	95% *CI*	*HR*	*p*	95% *CI*
Age	<0.001	(1.338–2.187)	1.535	0.001	(1.188–1.983)
Cr	<0.001	(1.288–2.051)	1.401	0.006	(1.102–1.783)
ALB	<0.001	(0.472–0.789)	0.654	0.002	(0.499–0.857)
PLT	0.001	(0.518–0.850)	0.779	0.049	(0.606–0.960)
LYR	0.087	(0.966–1.650)	1.325	0.037	(1.017–1.725)
ALT	0.025	(1.065–2.593)	1.716	0.019	(1.092–2.696)
Etiologies	0.061	(0.993–1.371)	1.144	0.132	(0.960–1.364)
FIB	0.010	(0.369–0.871)			
Hb	0.003	(0.501–0.865)			
Gender	0.078	(0.487–1.039)			

Cr, creatinine; ALB, albumin; PLT, platelet; LYR, lymphocyte ratio; Hb, hemoglobin; FIB, fibrinogen; ALT, alanine aminotransferase.

### Development of HHLWG-HPI and Internal Validation

**Figure 5:**
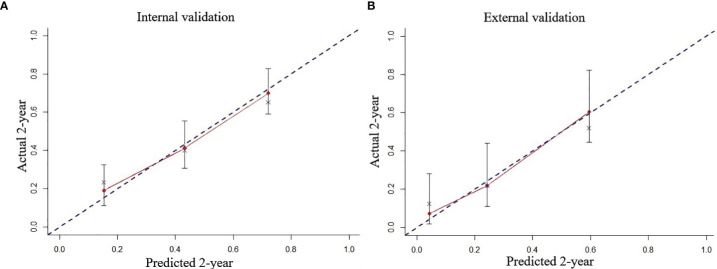
The predicted probability of 2-year OS by HHLWG-HPI was plotted on the x‐axis, and the actual 2-year OS was plotted on the y‐axis in internal **(A)** and external **(B)** validation.

**Figure 6:**
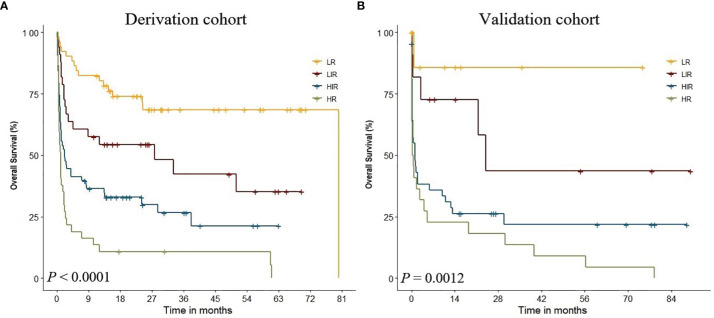
KM analysis of four risk groups in derivation **(A)** and validation **(B)** cohort by HHLWG-HPI.

In the multivariate Cox regression model, age was stratified into three groups (18–49, 50–65, and > 65 years); albumin in three groups (<29.8, 29.8–35.2, and >35.2 g/l); creatinine in three groups (<53, 53–66, and >66 μmol/l); lymphocyte ratio in two groups (≤45.8 and >45.8%); alanine aminotransferase in two groups (≤40 and >40), and platelet in two groups (≤72 and >72 × 10^9^/l). After rounding up the hazard ratios of the significant variables, the current HHLWG-HPI used 6 factors with a maximum of 9 scoring points (**Table 3**). Additionally, we assessed the accuracy using C-index and we also calculated the error of the model fitting on survival data using Brier Score in the internal validation. On average, the derivation cohort generated a high C-index (0.796) and a low brier score (0.184). The calibration curve for the probability of 5-year OS showed a good correlation between the actual observed outcome and the prediction by HHLWG-HPI (**Figure 5A**). Using this index, four risk groups were formed: low risk (LR, ≤3 pts), low-intermediate risk (LIR, 4 pts), high-intermediate risk (HIR, 5–6 pts), and high risk (HR, ≥7 pts) (**Figure 6A**). There was clearly a difference in OS between each of these risk groups (global comparison *p* < 0.001; LR vs. LIR *p* = 0.012; LIR vs. HIR *p* = 0.024; HIR vs. HR *p* = 0.014). This model showed precise stratification of outcomes with 5-year OS of 68.5%, 35.2%, 21.3%, and 10.8%, respectively.

**Table 3:** The HHLWG-HPI.

HHLWG-HPI	Score
Age (year)	
<50	0
50–65	1
>65	2
ALB (g/L)	
>35.2	0
29.8–35.2	1
<29.8	2
Cr (μmol/L)	
<53	0
53–66	1
>66	2
PLT (×10^9/L)	
≥72	0
<72	1
ALT (U/L)	
<40	0
≥40	1
LYR (%)	
<45.8	0
≥45.8	1

Alb, albumin; Cr, creatinine; PLT, platelet; ALT, alanine aminotransferase; LYR, lymphocyte ratio.

### External Validation of HHLWG-HPI

We further validated the HHLWG-HPI externally by the calibration curve and by computing the C-index and Brier Score in an independent validation cohort of 86 patients. The C-index was 0.758, and the Brier Score was 0.176 (**Figure 5B**). Noticeably, 25.58% of patients were classified as high risk. KM analysis also showed that there were significant differences in OS between the four groups, confirming the reproducibility of the HHLWG-HPI (**Figure 6B**).

### Validation in Retrospective Regimens With HHLWG-HPI

In this retrospective study, multiple regimens were adopted for adult HLH, and patients in the DEP regimen group had the best 5-year OS (62.5%). However, it was confusing that patients in the HLH-94 regimen group had poor survival (5-year OS 22.9%), even worse than that in steroid regimen group (5-y OS 30%), which was probably a consequence of improper individual treatment strategies. Thus, we used the HHLWG-HPI model to verify the whole cohort. Chi-square analysis results suggested that the proportion of HIR/HR patients in the HLH-94 regimen was significantly higher than that in the CHOP/CHOP-like regimen (*χ* ^2^ = 6.608, *p* = 0.010). Similarly, the proportion of HIR/HR patients in the supportive treatment group was higher than that in the HLH-2004 regimen (*χ* ^2^ = 5.955, *p* = 0.015) and CHOP/CHOP-like regimen (*χ* ^2^ = 17.161, *p* < 0.001). Due to the lack of accurate risk stratification, the individual therapeutic regimen could not be reasonably selected, resulting in the confusing survival data in different groups.

## Discussion

Hemophagocytic lymphohistiocytosis is a rare, life-threatening disorder with excessive immune activation. The therapeutic regimens available for the treatment of adult HLH vary widely in intensity (24, 25). Due to the lack of an accurate prognostic stratification system, there are no individualized therapeutic criteria for adult HLH (26, 27). Therefore, based on feasible clinical variables, we first established the HHLWG-HPI model, providing a novel stratification system for adult HLH.

Several studies have shown that albumin, age, and alanine aminotransferase can be independent prognostic factors for HLH (13, 28). Based on the results of this cohort study, KM analysis showed that etiologies were not independent factors for the prognosis of adult HLH. However, further analysis between subgroups found that the survival rate of patients in the infection-associated group was significantly higher than that in the tumor-associated group. Of 270 cases, EBV-DNA data were available in 163 evaluable patients, and KM analysis showed that a high EBV-DNA level was associated with poor outcome. We also attempted to explore the effect of ferritin on the prognosis of adult HLH, but approximately 36% data were missing, and there was no correlation between ferritin level and the prognosis of adult HLH. Although therapeutic regimens were not independent factors for the prognosis, the 5-year survival rate of the patients in the DEP regimen was higher than that in the HLH-94 regimen, HLH-04 regimen, and steroid regimen in this study. In addition, we proved that the levels of immune globulin were not associated with survival. However, patients with a low ratio of CD4/CD8 were with better prognosis.

Multivariate analysis showed that age, albumin, creatinine, alanine transaminase, lymphocyte ratio, and platelet were independent prognostic factors for adult HLH, and these variables were grouped by respective optimal cutoff points. X-Tile program results suggested that platelet >72 × 10^9^/l could predict a better outcome and older age at onset, and ALT ≥ 40 U/l and Cr>66 μmol/l were positively associated with poor survival, which were consistent with previous reports (26, 29). After the model iterations of multivariate analysis, we developed the HHLWG-HPI model with a maximum of score of 9 and 4 risk groups. The 5-year OS of the high-risk group in the derivation cohort was higher than that in the validation cohort (10.8% vs. 4.5%), and we found that the median age of patients in the validation population was higher, which may offer an explanation of a lower OS in the validation cohort. In addition, the derivation cohort generated a high C-index (0.703) and a low Brier Score (0.189), and the C-index and Brier Score were 0.721 and 0.169, respectively. It is worth noting that both cohorts are unselected and from real-world settings.

The HLH-94 regimen and the HLH-2004 regimen are still the current first-line treatments for HLH. Wang et al. showed that the DEP regimen was an optimal salvage therapy for adult HLH with an overall response of 76.2% (10), which is worth noting. In this real-world retrospective study, the DEP regimen showed the best clinical response, but HLH-94, HLH-2004 regimen, and supportive treatment groups showed poor clinical response. The differences in clinical response among treatment groups may be due to different risks of patients, so the HHLWG-IPI model was used to validate the whole cohort, and the results suggested that the proportion of HIR/HR patients in the HLH-94 regimen was significantly higher than that in the CHOP/CHOP-like regimen (**Table 4**). There were also a high proportion of HIR/HR patients in steroid regimen and antiviral regimen groups, but the 5-year OS of patients was even higher than that in the HLH-94 regimen group, which was worth further discussion in the following studies. In this study, all patients in the DEP regimen were classified into low-risk and low-intermediate-risk groups, indicating that the DEP regimen could also be used for the treatment of patients at low-risk and low-intermediate-risk groups. However, due to the limited sample size, further studies are needed.

**Table 4:** Distribution of patients in different therapeutic regimens groups.

Therapeutic regimens	Distinct risk groups				Total (%)
	LR (%)	LIR (%)	HIR (%)	HR (%)	
DEP regimen	1 (16.67)	5 (83.33)			6 (2.22)
CHOP/CHOP-like	9 (40.91)	7 (31.82)	2 (9.09)	4 (18.18)	22 (8.15)
Steroid	12 (24.00)	6 (12.00)	24 (48.00)	8 (16.00)	50 (18.52)
Antiviral	10 (16.67)	10 (16.67)	29 (48.33)	11 (18.33)	60 (22.22)
HLH-2004	8 (30.77)	5 (19.23)	10 (38.46)	3 (11.54)	26 (9.63)
HLH-94	9 (23.08)	6 (15.38)	17 (43.59)	7 (17.95)	39 (14.45)
Supportive treatment	11 (16.42)	5 (7.46)	25 (37.31)	26 (38.81)	67 (24.81)

In conclusion, based on multicenter data of HHLWG, we developed the HHLWG-HPI model, which will potentially provide criteria for accurate stratification and individual treatment strategy in adult HLH. However, in this retrospective study, we did not collect the data of genetic measurements, sCD25, and pro-inflammatory markers, which may reduce model sensitivity and specificity. Further prospective multicenter studies are urgently needed to validate the model.

## Data Availability Statement

The raw data supporting the conclusions of this article will be made available by the authors, without undue reservation.

## Ethics Statement

Study approval was obtained from the independent Ethics Committees of each participating center in HHLWG. The patients/participants provided their written informed consent to participate in this study.

## Author Contributions

WS, SH, and KX: designed this study. ZS and CH: analysis and interpretation. ZS, YJ, QS, SZ, XC, LH, YW, QL, HZ, XL, LW, JJ, YM, WG, FW, CW, YS, JY, TZ, CS, XS, LX, DY, HS, JC, DL, ZL, and ZW: acquisition of data. JC, DL, ZL, and ZW provided the advices of this study. All authors contributed to the article and approved the submitted version.

## Funding

This study was funded by the Natural Science Foundation of Jiangsu Province, Grant/Award Number: BK20171181; Jiangsu Key Research and Development Project of Social Development, Grant/Award Number: BE2019638; and Young Medical Talents of Jiangsu Science and Education Health Project, Grant/Award Number: QNRC2016791.

## Conflict of Interest

The authors declare that the research was conducted in the absence of any commercial or financial relationships that could be construed as a potential conflict of interest.

## Publisher’s Note

All claims expressed in this article are solely those of the authors and do not necessarily represent those of their affiliated organizations, or those of the publisher, the editors and the reviewers. Any product that may be evaluated in this article, or claim that may be made by its manufacturer, is not guaranteed or endorsed by the publisher.
